# Thymic atypical carcinoid tumors with elevated mitotic counts in a patient with multiple endocrine neoplasia: A case report

**DOI:** 10.1111/1759-7714.14863

**Published:** 2023-03-20

**Authors:** Shuntaro Hiro, Shuhei Teranishi, Tomoe Sawazumi, Satoshi Nagaoka, Chihiro Sugimoto, Hirokazu Nagayama, Wataru Segawa, Yukihito Kajita, Chihiro Maeda, Sousuke Kubo, Kenichi Seki, Ken Tashiro, Nobuaki Kobayashi, Masaki Yamamoto, Makoto Kudo, Takeshi Kaneko

**Affiliations:** ^1^ Respiratory Disease Center Yokohama City University Medical Center Yokohama Japan; ^2^ Division of Pathology Yokohama City University Medical Center Yokohama Japan; ^3^ Department of Pulmonology Yokohama City University School of Medicine Graduate School of Medicine Yokohama Japan

**Keywords:** atypical carcinoid tumors with elevated mitotic counts, large‐cell neuroendocrine carcinoma, multiple endocrine neoplasia, thymic neuroendocrine tumor

## Abstract

Thymic neuroendocrine tumors associated with multiple endocrine neoplasia are only defined as carcinoid and are not associated with large‐cell neuroendocrine carcinoma (LCNEC). We report the case of a multiple endocrine neoplasia type 1 patient with atypical carcinoid tumors with elevated mitotic counts (AC‐h), an intermediate condition between carcinoid and LCNEC. A 27‐year‐old man underwent surgery for an anterior mediastinal mass and was diagnosed with thymic LCNEC. Fifteen years later, a mass appeared at the same site, which was determined to be a postoperative recurrence based on the pathological results of a needle biopsy and the clinical course. The patient's disease remained stable for 10 months on anti‐programmed death‐ligand 1 antibody and platinum‐containing chemotherapy. The needle biopsy specimen was submitted for next‐generation sequencing, which revealed a *MEN1* gene mutation, and after further examination, a diagnosis of multiple endocrine neoplasia type 1 was made. A re‐examination of the surgical specimen from 15 years prior showed that it corresponded to AC‐h. Although thymic AC‐h is classified as thymic LCNEC according to the current definition, our data suggests that a search for multiple endocrine neoplasia is warranted in such patients.

## Graphical abstract

We report a case of thymic neuroendocrine tumors 15 years after thymic atypical carcinoid tumors with elevated mitotic counts surgery, and close examination revealed multiple endocrine neoplasia type 1. The patient underwent anti‐programmed death‐ligand 1 antibody and platinum‐containing chemotherapy and has maintained stable disease for 10 months. When a diagnosis of thymic atypical carcinoid tumors with elevated mitotic counts is performed, multiple endocrine neoplasia should be investigated.

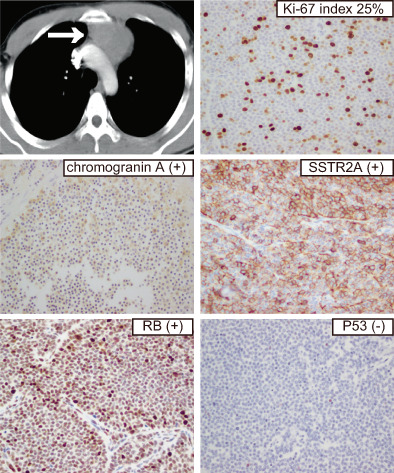

## INTRODUCTION

In the 2021 World Health Organization (WHO) classification of thymic tumors, thymic neuroendocrine tumors (TNET) are classified into four types: typical carcinoid, atypical carcinoid (AC), large‐cell neuroendocrine carcinoma (LCNEC), and small‐cell carcinoma (SCC).^1^ Recently, atypical carcinoid tumors with elevated mitotic counts (AC‐h), an intermediate condition between AC and LCNEC, have been identified.^1^, ^2^, ^3^ According to the current definition, TNET complicated patients with multiple endocrine neoplasia (MEN) is only carcinoid and is not associated with LCNEC.^1^ Here, we report a case of AC‐h in a patient with MEN type 1.

## CASE REPORT

**FIGURE 1:**
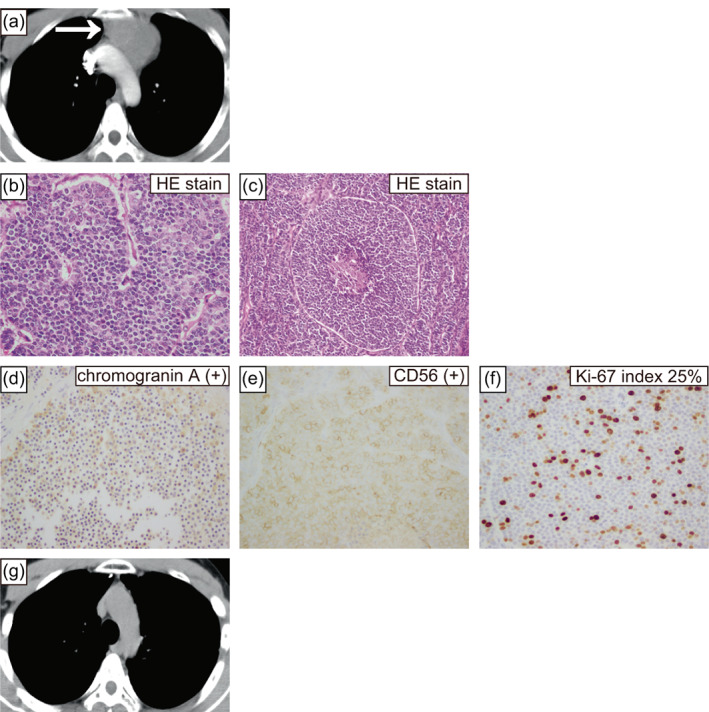
Computed tomography (CT) performed 15 years prior showed a mass in the anterior mediastinum (a). Microscopic findings of the surgical specimen from 15 years prior (hematoxylin–eosin staining and immunohistochemical findings, (b)–(f)). Hematoxylin–eosin staining findings comprised relatively homogeneous cells with weak atypia (b), (c). Hematoxylin–eosin staining revealed >10 mitotic counts at 2 mm^2^ (b) and necrosis (c). Immunohistochemical staining was positive for chromogranin A (d) and CD56 (e). The Ki‐67 index was 25% (f). CT images taken 9 years ago (6 years after surgery) showed that the mass in the anterior mediastinum had disappeared (g). The magnification was 400× (b)–(f). HE, hematoxylin and eosin.

A 27‐year‐old male presented to our clinic with a chest X‐ray showing an enlarged mediastinum. Computed tomography (CT) showed a mass in the anterior mediastinum (Figure 1(a)) with no evidence of distant or lymph node metastasis, and surgery was performed. Pathological findings comprised relatively homogeneous cells with weak atypia (Figure 1(b),(c)), and immunostaining was positive for chromogranin A and CD56 (Figure 1(d),(e)). Mitotic counts exceeded 10 cells at 2 mm^2^ (Figure 1(b)) with necrosis (Figure 1(c)) and a Ki‐67 index of 25% (Figure 1(f)), and a diagnosis of thymic LCNEC was made. The patient received postoperative radiation (50 Gray/25 fractions) and four courses of chemotherapy (cisplatin 80 mg/m^2^ body surface area and etoposide 100 mg/m^2^ body surface area). The patient had no recurrence on CT for 6 years postoperatively (Figure 1(g)) and was placed on an annual chest X‐ray as follow‐up.

**FIGURE 2:**
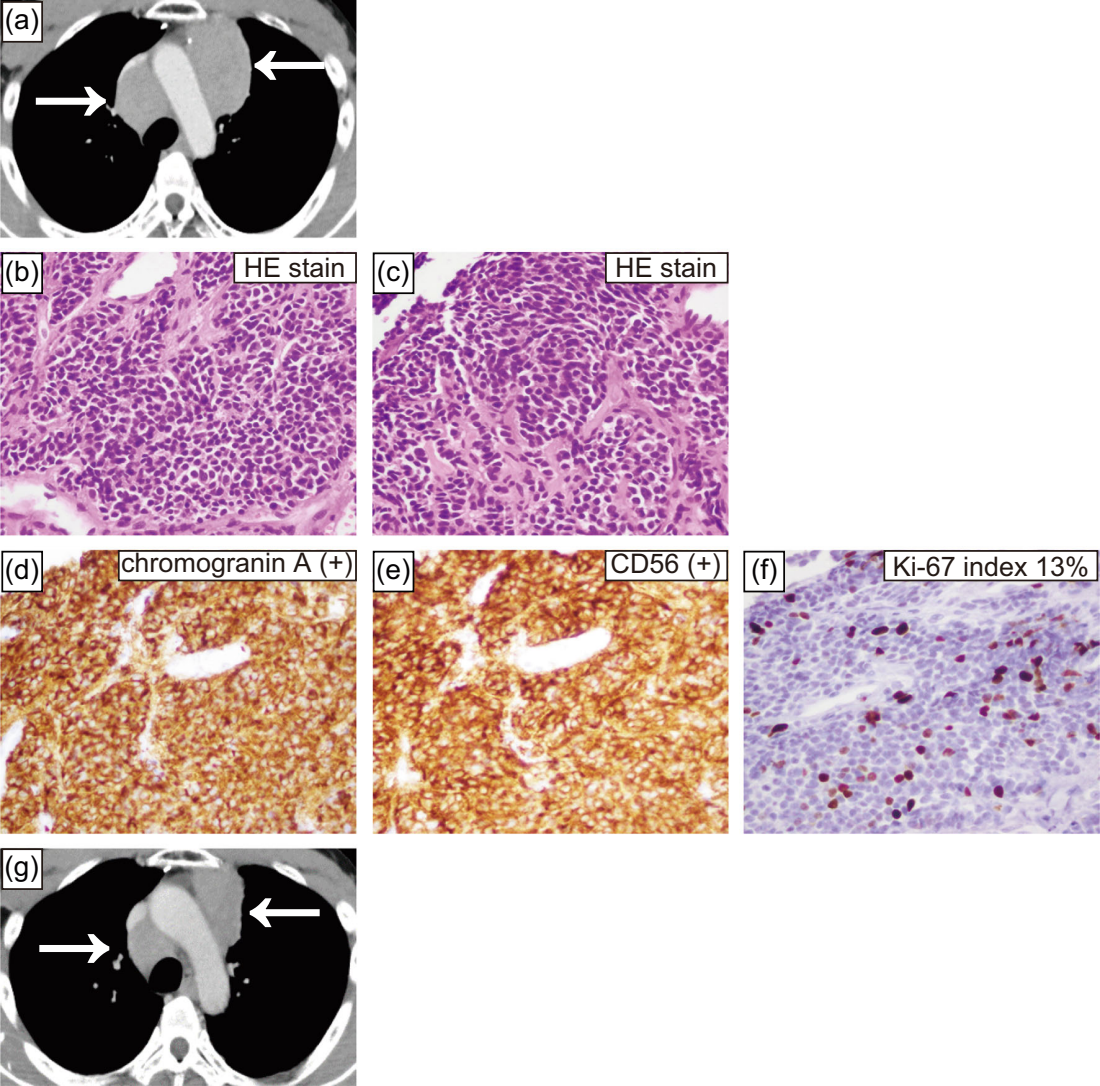
Computed tomography (CT) taken for the current case again showed a mass in the anterior mediastinum (a). Microscopic findings of this case's needle biopsy specimen (hematoxylin–eosin staining and immunohistochemical findings, (b)–(f)). Hematoxylin–eosin staining findings comprised relatively homogeneous cells with weak atypia (b), (c). Hematoxylin–eosin staining showed one mitotic count at 2 mm^2^ (b) and no necrosis (c). Immunohistochemical staining was positive for chromogranin A (d) and CD56 (e). The Ki‐67 index was 13% (f). CT demonstrating the best response to first‐line chemotherapy showing shrinkage within the stable disease range (g). The magnification was 400× (b)–(f). HE, hematoxylin and eosin.

Fifteen years later, he was again diagnosed with mediastinal enlargement on a chest X‐ray. CT showed a mass in the anterior mediastinum (Figure 2(a)), and a CT‐guided needle biopsy was performed. Pathological findings comprised relatively homogeneous cells with weak atypia (Figure 2(b),(c)), and immunostaining was positive for chromogranin A and CD56 (Figure 2(d),(e)). Mitotic counts were one at 2 mm^2^ (Figure 2(b)), no necrosis was observed (Figure 2(c)), and the Ki‐67 index was 13% (Figure 2(f)), indicating thymic carcinoid pathology. However, because the needle biopsy specimen may not reflect the whole tumor and the mass occurred at the same site as 15 years ago, we concluded that it was a postoperative recurrence of thymic LCNEC. Radical resection was difficult because of the spread of the mass, and re‐radiation was also difficult because of the patient's history of radiation. The patient was treated with first‐line cisplatin (80 mg/m^2^ body surface area), etoposide (100 mg/m^2^ body surface area), and durvalumab (1500 mg/body) for four courses, followed by durvalumab maintenance therapy, as in extensive‐stage lung SCC.

Thymic LCNEC is rare cancer, and a needle biopsy specimen was submitted to FoundationOne CDx (Foundation Medicine) for next‐generation sequencing, which showed *MEN1* and CCCTC‐binding factor gene mutations was found. A blood test for the *MEN1* gene revealed the same variant. After careful examination, the patient had primary hyperparathyroidism and was diagnosed with MEN type 1. The TNETs 15 years prior and in this case were determined to have occurred based on MEN type 1. It was difficult to determine whether the current TNET was a postoperative recurrence or a new occurrence. The patient has maintained tumor shrinkage in a stable disease range for 10 months in response to first‐line chemotherapy (Figure 2(g)), and is scheduled to continue durvalumab until the disease progresses.

## DISCUSSION

TNETs are rare diseases, accounting for ~5% of all mediastinal and thymic tumors and 0.4% of all neuroendocrine tumors.^4^, ^5^, ^6^ In the 2021 WHO classification of thymic tumors, TNETs are classified into four types: low‐grade typical carcinoid, intermediate‐grade AC, high‐grade LCNEC, and SCC.^1^ They are classified according to morphological characteristics, mitotic counts, and necrosis and the Ki‐67 index is also used as a reference.

**FIGURE 3:**
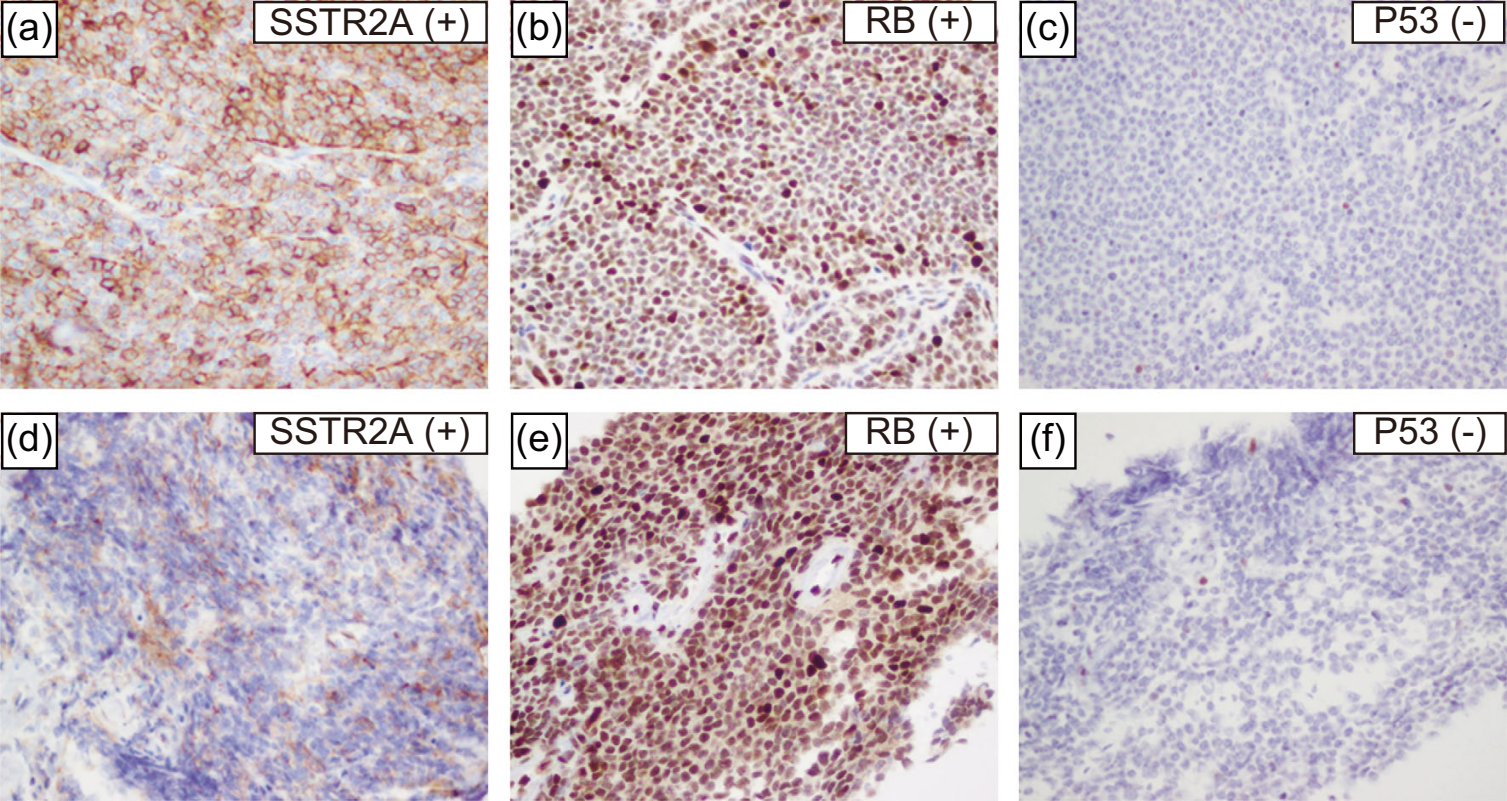
Microscopic findings of the surgical specimen from 15 years prior (a)–(c). Immunohistochemical staining was positive for somatostatin receptor 2a (a) and RB transcriptional corepressor 1 (b) and negative for tumor protein p53 (c). Microscopic findings of this case's needle biopsy specimen (d)–(f). Immunohistochemical staining was positive for somatostatin receptor 2a (d) and RB transcriptional corepressor 1 (e) and negative for tumor protein p53 (f). The magnification was 400× (a)–(f). P53, tumor protein p53; RB, RB transcriptional corepressor 1; SSTR2A, somatostatin receptor 2a.

Recently, the existence of AC‐h with carcinoid morphological features and elevated mitotic counts and Ki‐67 index has been reported.^1^, ^2^, ^3^ Under the current definition, thymic AC‐h is classified as a thymic LCNEC, but may be added as a new group of TNETs in the future.^1^ Immunohistochemical markers that distinguish thymic AC‐h from thymic LCNEC include chromogranin A, somatostatin receptor 2a (SSTR2A), RB transcriptional corepressor 1 (RB), and tumor protein p53 (P53).^1^, ^2^ By the current definition, the surgical specimens from 15 years ago are now classified as thymic LCNEC, but are positive for chromogranin A, SSTR2A, RB, and P53 negative (Figure 1(d) and Figure 3(a)–(c)), which were considered to correspond to thymic AC‐h. The same findings were observed in the needle biopsy specimen from the current tumor (Figure 2(d) and Figure 3(d)–(f)). According to the current definition, TNET complicated patients with MEN are only carcinoid and are not associated with LCNEC.^1^ However, when thymic AC‐h is diagnosed, as in this case, searching for MEN is necessary.

A treatment option for advanced or unresectable thymic carcinoid is everolimus, a mammalian target of rapamycin inhibitor.^7^, ^8^ However, there are few studies with a high level of evidence for the treatment of thymic LCNEC, and the treatment is similar to that for lung SCC.^9^, ^10^, ^11^ The patient underwent anti‐programmed death‐ligand 1 antibody and platinum‐containing chemotherapy according to the recommendations for extensive‐stage lung SCC,^12^, ^13^ and has maintained stable disease for about 10 months.

In this case, TNET occurred 15 years after thymic AC‐h surgery, and close examination revealed MEN type 1. Immunostaining for chromogranin A, SSTR2A, RB, and P53 should be performed to differentiate thymic AC‐h from thymic LCNEC. MEN should be investigated when thymic AC‐h is diagnosed, using next‐generation sequencing and other methods. Therefore, further studies are needed to determine whether everolimus or anti‐programmed death‐ligand 1 antibody and platinum‐containing chemotherapy should be preferred for thymic AC‐h.

## AUTHOR CONTRIBUTIONS

S.T. and S.H: Writing ‐ Original Draft. T.S., S.N., C.S., H.N., W.S., Y.K., C.M., S.K., K.S., K.T: Writing ‐ Review & Editing. N.K., M.Y., M.K., T.K: Visualization and Supervision. All authors approved the final version of the manuscript to be published.

## CONFLICT OF INTEREST

The authors have no conflicts of interest to declare.
